# The value of high-resolution computed tomography (HRCT) to determine exercise ventilatory inefficiency and dynamic hyperinflation in adult patients with cystic fibrosis

**DOI:** 10.1186/s12931-019-1044-8

**Published:** 2019-04-24

**Authors:** Ernesto Crisafulli, Elisabetta Teopompi, Silvia Luceri, Francesco Longo, Panagiota Tzani, Paolo Pagano, Antonella Ielpo, Chiara Longo, Marcello Di Paolo, Nicola Sverzellati, Paolo Palange, Alfredo Chetta, Giovanna Pisi

**Affiliations:** 1 Department of Medicine and Surgery, Respiratory Disease and Lung Function Unit, University of Parma, Via Rasori 10, 43126 Parma, Italy; 2 Section of Radiology, Unit of Surgical Sciences, Department of Medicine and Surgery, University of Parma, Parma, Italy; 3 Cystic Fibrosis Unit, University Hospital of Parma, Parma, Italy; 4 Department of Public Health and Infectious Diseases, Sapienza University of Rome, Rome, Italy

**Keywords:** Cystic fibrosis, Ventilatory inefficiency, Dynamic hyperinflation, Brody II score

## Abstract

**Introduction:**

In Cystic Fibrosis (CF), exercise ventilatory inefficiency and dynamic hyperinflation (DH) cause exercise limitation and induce poor exercise tolerance. High-resolution computed tomography (HRCT) of the lung can detect pulmonary abnormalities in CF patients. We aimed to identify the determinants of exercise ventilatory inefficiency and DH using HRCT-derived metrics.

**Methods:**

Fifty-two adult CF patients were prospectively enrolled; all participants underwent cardio-pulmonary exercise test (CPET) and HRCT. Radiological impairment was evaluated by the Brody II scoring system. Slope and intercept of the minute ventilation/CO_2_ production (V’_E_/V’_CO2_) regression line and the ratio of inspiratory capacity/total lung capacity (IC/TLC) at rest and at peak of exercise were measured.

**Results:**

Four groups of patients were identified based on the combination of ventilatory efficiency (*Vef*) or inefficiency (*Vin*) and the presence/absence of DH. Compared to other groups, CF adults with *Vin* and DH had worse functional status and higher total (T), bronchiectasis (B) and air trapping (AT) scores at HRCT. Significant correlations were found between V’_E_/V’_CO2 intercept_ and V’_E_/V’_CO2 slope_ (ρ − 0.455, *p* = 0.001) and between V’_E_/V’_CO2 intercept_ and Δ inspiratory capacity (IC) (ρ − 0.334, *p* = 0.015*).* Regression analysis identified AT score (cut-off 7.9, odds ratio-OR 3.50) as the only independent predictor of V*in* and T (cut-off 53.6, OR 4.98), B (cut-off 16.1, OR 4.88), airways wall thickening (AWT) (cut-off 13, OR 3.41), and mucous plugging (MP) scores (cut-off 11.7, OR 4.18) as significant predictors of DH.

**Conclusion:**

In adult CF cohort, values of HRCT metrics are determinants of *Vin* (AT) and DH (T, B, AWT, MP).

## Introduction

In Cystic Fibrosis (CF) patients, pulmonary infections lead to a progressive decline in lung function [1]. Although spirometry and, particularly, the forced expiratory volume at 1st second (FEV_1_) is considered a valuable measure for lung function monitoring [2], variables derived from exercise testing, such as the cardio-pulmonary exercise test (CPET), are more sensitive than spirometry in detecting early structural pulmonary changes in CF patients [3].

In adolescents with CF, an exaggerated ventilatory response to exercise but adequate to V’_CO2_ (normal V’_E_/V’_CO2 slope_) was documented [4] and, interestingly, in adult CF patients the V’_E_/V’_CO2_ at peak of exercise was an independent determinant of exercise limitation, especially in those with a worse lung function [5]. Moreover, in adults with CF, a high prevalence of exercise dynamic hyperinflation (DH) was found, secondary to the development of air trapping during exercise; DH was also shown to be associated to poor resting lung function, reduced exercise tolerance and increased exertional dyspnea [6].

High-resolution computed tomography (HRCT) of the lung is able to demonstrate a broad range of pulmonary abnormalities in CF patients with mild-to-moderate lung disease [7], identifying lower airway inflammation and early lung impairments [8]. The Brody score [7] is a composite HRCT score including different radiological features of lung disease (Bronchiectasis-B, Airways Wall Thickening-AWT, Mucous plugging-MP, Parenchyma-P and Air Trapping-AT) and demonstrating a high inter-observer reproducibility [9]. In CF patients, higher Brody scores indicate more severe disease [7]; they are also strongly associated with lung disease progression [10], predicting the rate of pulmonary exacerbations up to 10-year follow-up [11].

Interestingly, with regards to the relationship between HRCT-derived variables and the ventilatory response to exercise, the emphysema HRCT metrics were independent predictors of exercise-induced DH and V’_E_/V’_CO2 slope_ in COPD patients [12]. In CF adults, however, no studies assessed the value of the variables derived from HRCT to determine both the exercise ventilatory response to CO_2_ and DH. Therefore, we aimed to identify in a large cohort of adult patients with CF the determinants of exercise ventilatory response to CO_2_ and DH using HRCT-derived metrics, as expressed by the Brody II score.

## Methods

### Patients

We conducted this prospective study at the University Hospital of Parma (Italy) between June 2013 and July 2017. The main inclusion criteria were a confirmed diagnosis of CF as recommended by international guidelines [13], age > 18 years and a stable respiratory condition (i.e. patients free from exacerbations) for at least 4 weeks before enrolment Patients with lung transplant, pregnancy and concomitant malignancies or comorbidities (i.e. heart failure, previous rib fracture) which could preclude their safety were excluded.

The study was conducted according to the Declaration of Helsinki; all the procedures and their risks were explained to the patients, who gave their written informed consent for the participation to the study. The ethical committee of the University Hospital of Parma approved the protocol (approval number: 200084; 07 June 2013). All participants’ data were anonymously analyzed and reported. No extramural funding was used to support the study.

### Measurements

For all patients, anthropometric characteristics (age, gender, body mass index-BMI and fat-free mass index-FFMI), presence of diabetes, CFTR mutations (F508del/F508del, F508del/Other, or Other/Other), *Pseudomonas aeruginosa* colonization, long-term oxygen therapy and domiciliary non-invasive mechanical ventilation were recorded at the enrolment. BMI was calculated in kilograms per square meter. Body composition was also assessed by a foot-to-foot bioelectrical impedance analysis (BIA) method by a body composition analyzer (model SC-331S, Tanita, Tokyo, Japan). The fat-free mass (FFM) was standardized for height to calculate the FFM index (FFMI = FFM/height squared, in kilograms per square meter).

All lung function tests were performed according to the international recommendations [14]. A flow-sensing spirometer and a body plethysmograph connected to a computer for data analysis (Vmax 22 and 6200, Sensor Medics, Yorba Linda, USA) were used for the measurements. FEV_1_ and forced vital capacity (FVC) were recorded; the FEV_1_/FVC ratio was considered as the index of airflow obstruction. Thoracic gas volume (TGV) was measured by body plethysmography with the patients painting against a closed shutter at a frequency slightly < 1 Hz and supporting their cheeks with their hands. Total lung capacity (TLC) was obtained as the sum of TGV and related inspiratory capacity (IC). To ensure reproducibility for each spirometry and lung volume variables at least three measurements were taken; the highest value was considered. The flow-sensor was calibrated before each test using a three-liter syringe. FEV_1_, FVC, and IC were expressed as a percentage of the predicted values [15].

Incremental CPET was performed according to the standardized international procedure [16]. Patients were not pre-treated with β_2_ agonists before testing, but they could take their current therapies. After calibrating the oxygen and carbon dioxide analysers and flow mass sensor, patients were invited to sit on an electromagnetically braked cycle ergometer (Corival PB, Lobe Bv, Groningen, The Netherlands) and the saddle was adjusted properly to avoid the maximal extension of the knee. The exercise protocol involved an initial rest of 3 min, followed by unloaded cycling for another 3 min with an increment of 5 to 15 watts every minute, according to the patient’s anthropometry and degree of functional impairment, in order to achieve an exercise time between 8 and 12 min. Patients were invited to maintain a pedalling frequency of 60 rates per minute.

Breath-by-breath oxygen uptake (V’_O2_ in L/min), carbon dioxide production (V’_CO2_ in L/min), tidal volume (V_T_ in L) and minute ventilation (V’_E_ in L/min) were recorded during the test (CPX/D; Med Graphics, St Paul, MN, USA). Patients were continuously monitored with a 12-lead electrocardiogram (Welch Allyn CardioPerfect, Delft, the Netherlands) and a pulse oximeter (Pulse Oximeter 8600, Nonin Medical Inc., MPLS, Mn U.S.A.). Blood pressure was measured every two minutes. Stopping criteria consisted of symptoms, such as unsustainable dyspnea, leg fatigue or chest pain, a significant ST-segment depression at ECG, or a drop in systolic blood pressure or oxygen saturation ≤ 84% [17].

Workload and V’_O2 at peak_ were recorded as the mean value of watts and V’_O2_ during the last 20 s of the test. V’_O2_ at peak was expressed as mL/kg/min. The ventilatory response during exercise was expressed as a linear regression function by plotting V’_E_ against V’_CO2_ obtained every 10 s, excluding data above the ventilatory compensation point [17]. Then, the slope and Y intercept values were obtained from the V’_E_/V’_CO2_ regression line. V’_E_/V’_CO2_ at rest_,_ V’_E_/V’_CO2 slope_ and V’_E_/V’_CO2 intercept_ (L∙min^− 1^) were recorded. Values of V’_E_/V’_CO2 slope_ ≥ 30 and < 30, derived from the median value in our cohort, were used to define patients with an exercise ventilatory inefficiency (V*in*) and patients with ventilatory efficiency (V*ef*), respectively [18].

The end-tidal pressure of CO_2_ (P_ETCO2_, in mm Hg) was measured as the mean of P_ETCO2_ during the 3-min rest period and during the last 20 s of the test; moreover, the difference between P_ETCO2_ at peak and P_ETCO2_ at rest (Δ P_ETCO2_) was recorded.

Changes in operational lung volumes were assessed every two minutes during exercise and at peak exercise, taking the IC measured at rest, as the baseline. After a full explanation of the procedure, satisfactory technique and reproducibility of IC maneuvers were established during an initial practice session at rest. Assuming that TLC remains constant during exercise [19], a change in IC (Δ IC, L) ≤ − 0.100 L at peak of exercise was considered to define patients with DH, as reported from the literature [20].

The cardiovascular response to exercise was expressed by the following parameters: oxygen pulse (O_2_ pulse), HR _recovery_ and double product (DP) reserve. O_2_ pulse (in mL/beats/min) was calculated by dividing instantaneous V_O2_ by the HR and was recorded at rest and as the change between the peak of exercise and the resting phase (Δ O_2_ pulse) [17]. HR _recovery_ (in beats) was defined as the reduction of the HR at the peak exercise level compared to the HR after one minute of the exercise cessation [17]. DP (mmHg*bpm) at rest and at maximal exercise was calculated by the product of systolic blood pressure and HR [21]; DP reserve was calculated as DP at maximal exercise minus DP at rest and was also expressed as a ratio to workload in watts.

Oxygen saturation (SpO_2_) was reported at rest and as the difference between values at peak of exercise and at rest (Δ SpO_2_). Peak dyspnea and fatigue were described as visual analogic scale (VAS) with 0–100 score and then divided by peak workload (watts) for analysis.

Patients were scanned with a 128-slice Somatom Definition Flash scanner (Siemens Medical Solutions, Forchheim, Germany). Technical parameters were as follows: volumetric acquisition, collimation 128 × 2 × 0.6 mm; 1 mm slice thickness, rotation time 0.28 msec; tube voltage 80–100 kVp; tube current modulation (CareDose) on. Images were visually scored using a window setting (− 1550 W, − 600 L). Each HRCT scan was evaluated independently by two radiologists (S.L. and P.P.), both with a 3-year imaging experience. Readers scored HRCTs using the Brody II scoring system [7]. The total score (T) is the sum of the following sub-scores: Bronchiectasis-B, Airways Wall Thickening-AWT, Mucous Plugging-MP, Parenchyma-P (e.g. bullae, ground glass and consolidation) and Air Trapping-AT with decreased attenuation areas. Each of the five sub-scores is rated according to presence and severity, in each of the six lobes, lingula being considered as a separate lobe. All scores and sub-scores are expressed as a percentage of the maximum possible score, ranging from 0 (no pathological findings) to 100 (maximum severity).

### Statistical analysis

Analyses were performed with IBM SPSS Statistics 25.0 (Armonk, New York, USA) and Shapiro-Wilk test was used to assess the normality of distribution. Data were reported as medians (1st quartile; 3rd quartile) for continuous variables with non-normal distribution or means ± standard deviation (SD) for those with normal distribution. Number of patients (%) was used for categorical variables. Comparison analysis were performed using the chi-square (*X*^2^) or the Fisher exact test for categorical variables and the *t* test or the nonparametric Mann-Whitney test for continuous variables. For multiple comparisons, the Bonferroni post-hoc analysis, the ANOVA, and the nonparametric Kruskal-Wallis test were used.

The interrater reliability was evaluated by a two-way random-effects model with mean of *k* raters and definition of consistency [22]. The intraclass correlation coefficients (ICC) were interpreted by the following scale: ICC values < 0.50 are indicative of “poor reliability”, values between 0.50 and 0.75 indicate “moderate reliability”, values between 0.75 and 0.90 indicate “good reliability”, and values greater than 0.90 indicate “excellent reliability” [23].

For correlation analysis, the Pearson or Spearman correlation coefficients were used for linear or normally distributed variables and for not linear or not normally distributed variables, respectively.

Receiver operating characteristic (ROC) curves were generated to calculate the area under the curve (AUC) with 95% confidence interval (CI) and to find the best cut-off with the related sensibility and specificity. Univariate and multivariate regression logistic models (method: Stepwise) were performed to predict the probability to have V*in* and DH (set as dependent variables). Odds ratios (OR) and 95% CI were calculated. A *p* value at two tails < 0.05 was considered significant.

## Results

Our study sample consisted of 52 adult patients with CF, normal-weight, with a wide range of lung function from normal to severe airflow obstruction and a normal-to-mildly reduced maximal exercise capacity. Variables related to spirometry and CPET demonstrated that, in comparison to patients with V*ef* and without DH and patients with V*in* and without DH, patients with V*in* and with DH had statistically significant lower values of FEV_1_% pred., FVC % pred., FEV_1_/FVC %, and lower values in V’_O2 at peak_ ml/kg/min and workload in watts at peak without reaching statistical significance. Table 1 summarises the results about anthropometric and lung function characteristics and Table 2 CPET-related variables.

**Table 1:** General characteristics according to the ventilatory efficiency and dynamic hyperinflation

Variables	Study sample *n = 52*	Patients with V*ef* and without DH *n = 10 (19%)*	Patients with V*in* and without DH *n = 13 (25%)*	Patients with V*ef* and with DH *n = 14 (27%)*	Patients with V*in* and with DH *n = 15 (29%)*	*p* value ^a^
Age, years	29.1 ± 8.1	30.9 ± 10.1	29.8 ± 8	26.3 ± 6	30 ± 8.7	0.494
Male, %	58	80	46	50	60	0.373
BMI, kg/m^2^	21.4 [19.5; 22.5]	22.3 [21.7; 24.3]	20.8 [19; 22.8]	20.9 [19.8; 21.9]	20.9 [18.8; 23.3]	0.230
FFMI, kg/m^2^	17.1 [15.4; 18]	17.6 [15.7; 18.3]	16.4 [14.5; 17.4]	17 [15.6; 18.2]	17.4 [15.3; 18]	0.402
Diabetes, %	31	50	23	29	27	0.523
CFTR mutations, %						0.972
F508del/F508del	42	40	39	36	54	
F508del/Other	41	40	46	43	33	
Other/Other	17	20	15	21	13	
*Pseudomonas aeruginosa* colonization, %	94	80	92	100	100	0.133
LTOT, %	8	0	0	14	13	0.328
Domiciliary NIMV, %	4	0	0	7	7	0.647
FEV_1_, % pred.	69.2 ± 22.3	85.2 ± 22.3	82.1 ± 20	61.7 ± 17.4 ^b c^	54.3 ± 15.6 ^b c^	**< 0.001**
FVC, % pred.	89.6 ± 18.3	98.6 ± 17.1	100.4 ± 20	86 ± 12.8	77.5 ± 14.1 ^b c^	**0.001**
FEV_1_/FVC, %	64.8 ± 12.2	72.8 ± 9.1	70.1 ± 11.8	59.9 ± 11.1 ^b^	59.6 ± 11.3 ^b^	**0.005**
IC at rest, % pred.	77.5 [61.5; 91.7]	76.5 [69.2; 104.5]	76 [57.5; 103]	78 [63.7; 89.5]	81 [57; 87]	0.977
V_T_ at rest, L	0.64 [0.48; 0.84]	0.61 [0.37; 0.71]	0.86 [0.67; 0.95] ^b^	0.50 [0.44; 0.81] ^c^	0.61 [0.54; 0.71] ^c^	**0.015**
RR at rest, breath/min	17.5 [13; 21.5]	18 [16.6; 20.7]	13 [11.8; 18]	17 [11.7; 24]	20 [15.9; 26]	0.510

Ventilatory efficiency (V*ef*) and inefficiency (V*in*) were defined according to the V’_E_/V’_CO2 slope_ < 30 and V’_E_/V’_CO2 slope_ ≥ 30, respectively. Patients without and with dynamic hyperinflation (DH) were defined according to the peak-to-rest change of IC (Δ IC, L) > − 0.100 L and ≤ − 0.100 L.

Data are shown as number of patients (%), means ± SD or medians [1st quartile; 3rd quartile], unless otherwise stated

*Abbreviations: BMI* body mass index, *FFMI* fat-free mass index, *LTOT* long-term oxygen therapy, *NIMV* non-invasive mechanical ventilation, *FEV*_*1*_ forced expiratory volume at 1st second, *FVC* forced vital capacity, *IC* inspiratory capacity, *V*_*T*_ tidal volume, *RR* respiratory rate

^a^*p*-value calculated between all groups; ^b^ *p*-value < 0.05 versus patients with V*ef* and without DH; ^c^ *p*-value < 0.05 versus patients with V*in* and without DH

Boldface variables are statistically significants

**Table 2:** CPET-related variables

Variables	Study sample *n = 52*	Patients with V*ef* and without DH	Patients with V*in* and without DH	Patients with V*ef* and with DH	Patients with V*in* and with DH	*p* value ^a^
V’_O2 at peak_, ml/kg/min	28.1 ± 8.2	31.0 ± 7.7	25.8 ± 7.3	30.7 ± 9.2	25.7 ± 7.9	0.180
Workload, watts	121 [97; 169]	168 [121; 216.5]	109 [98; 146.5]	127.5 [94.5; 188]	102 [88; 138]	0.067
Δ IC, L	− 0.19 [− 0.43; 0.15]	0.24 [− 0.05; 0.37]	0.16 [0.08; 0.55]	−0.43 [− 0.54; − 0.21] ^c e^	−0.38 [− 0.43; − 0.26] ^c e^	**< 0.001**
Δ V_T_, L	0.89 [0.51; 1.33]	1.34 [0.95; 1.61]	0.92 [0.44; 1.24]	0.88 [0.57; 1.19] ^b^	0.64 [0.29; 0.89] ^b^	**0.039**
RR at peak, breath/min	37 [32; 44]	34 [32.1; 41.5]	31 [28; 38.9]	38.8 [31.7; 42.5]	44 [36; 51]	0.073
V’_E_/V’_CO2 at rest_	43.4 ± 5.9	39.8 ± 5.1	42.1 ± 5.0	43.4 ± 5.3	46.9 ± 6.2 ^b^	**0.017**
V’_E_/V’_CO2 slope_	29.3 ± 4.8	25.6 ± 1.9	33.0 ± 4.4 ^c^	24.9 ± 2.7 ^e^	32.5 ± 2.3 ^c g^	**< 0.001**
V’_E_/V’_CO2 intercept_, L∙min^− 1^	3.28 [1.95; 4.33]	3.3 [2.5; 3.6]	0.8 [0.2; 2.6] ^b^	4 [3.2; 7.4] ^b d^	3.6 [2.6; 5] ^d^	**0.001**
O_2_ pulse at rest, mL/beat/min	3.2 [2.3; 3.9]	3.3 [2.1; 4.5]	3.3 [2.1; 3.9]	3 [2.5; 3.9]	3.3 [2.3; 3.9]	0.948
Δ O_2_ pulse, mL/beat/min	7.0 ± 2.6	8.8 ± 2.7	6.0 ± 2.1 ^b^	7.9 ± 3.0	5.9 ± 1.5 ^b^	**0.008**
Δ P_ETCO2_	6 [4; 9]	6.5 [4.7; 8]	3 [0.5; 6]	10 [6.7; 14] ^b e^	6 [3; 7] ^f^	**0.001**
HR _recovery_	23 [15.2; 28]	25 [21.7; 31]	23 [15.5; 27.5]	20.5 [14.2; 24.5]	23 [13; 29]	0.261
HR/V_O2_	56 [46; 65.7]	47.5 [41.7; 59]	59 [47.5; 74]	50 [42; 64.2]	62 [52; 73]	0.122
DP reserve	15,251.1 ± 4192.6	15,387.0 ± 5090.4	14,966.5 ± 3391.4	15,063.2 ± 4187.2	15,582.7 ± 4582.2	0.980
DP reserve/Workload	123.8 ± 35.8	97.1 ± 36.1	129.9 ± 36.3	117.4 ± 27.9	142.3 ± 31.9 ^b^	**0.011**
SpO_2_ at rest, %	96.7 ± 1.7	97.3 ± 1.9	97.5 ± 1.3	96.4 ± 1.8	95.9 ± 1.7	0.079
Δ SpO_2_, %	−2.0 ± 3.3	−0.6 ± 2.5	−1.1 ± 2.7	−3.9 ± 3.8	− 2.0 ± 3.4	0.065
Peak dyspnea	0.51 [0.39; 0.77]	0.43 [0.25; 0.62]	0.55 [0.44; 0.73]	0.52 [0.36; 0.88]	0.57 [0.45; 0.95]	0.384
Peak fatigue	0.65 [0.45; 0.82]	0.47 [0.38; 0.77]	0.74 [0.60; 0.88]	0.60 [0.34; 0.77]	0.71 [0.57; 0.91]	0.198

Data are shown as number of patients (%), means ± SD or medians [1st quartile; 3rd quartile], unless otherwise stated

Variables with Δ were calculated as peak-to-rest changes

*Abbreviations: V’*_*O2*_ oxygen uptake, *IC* inspiratory capacity, *V*_*T*_ tidal volume, *RR* respiratory rate, *V’*_*E*_ minute ventilation, *V’*_*CO2*_ carbon dioxide output, *P*_*ETCO2*_ end-tidal pressure of CO_2_, *HR* heart rate, *DP* double product, *SpO*_*2*_ oxygen saturation

Peak dyspnea and peak fatigue were described as visual analogic scale (VAS) with 0–100 score and peak workload ratio

^a^*p*-value calculated between all groups

^b^*p*-value < 0.05 versus patients with V*ef* and without DH

^c^*p*-value < 0.001 versus patients with V*ef* and without DH

^d^*p*-value < 0.05 versus patients with V*in* and without DH

^e^*p*-value < 0.001 versus patients with V*in* and without DH

^f^*p*-value < 0.05 versus patients with V*ef* and with DH

^g^*p*-value < 0.001 versus patients with V*ef* and with DH

Boldface variables are statistically significants

**Fig. 1:**
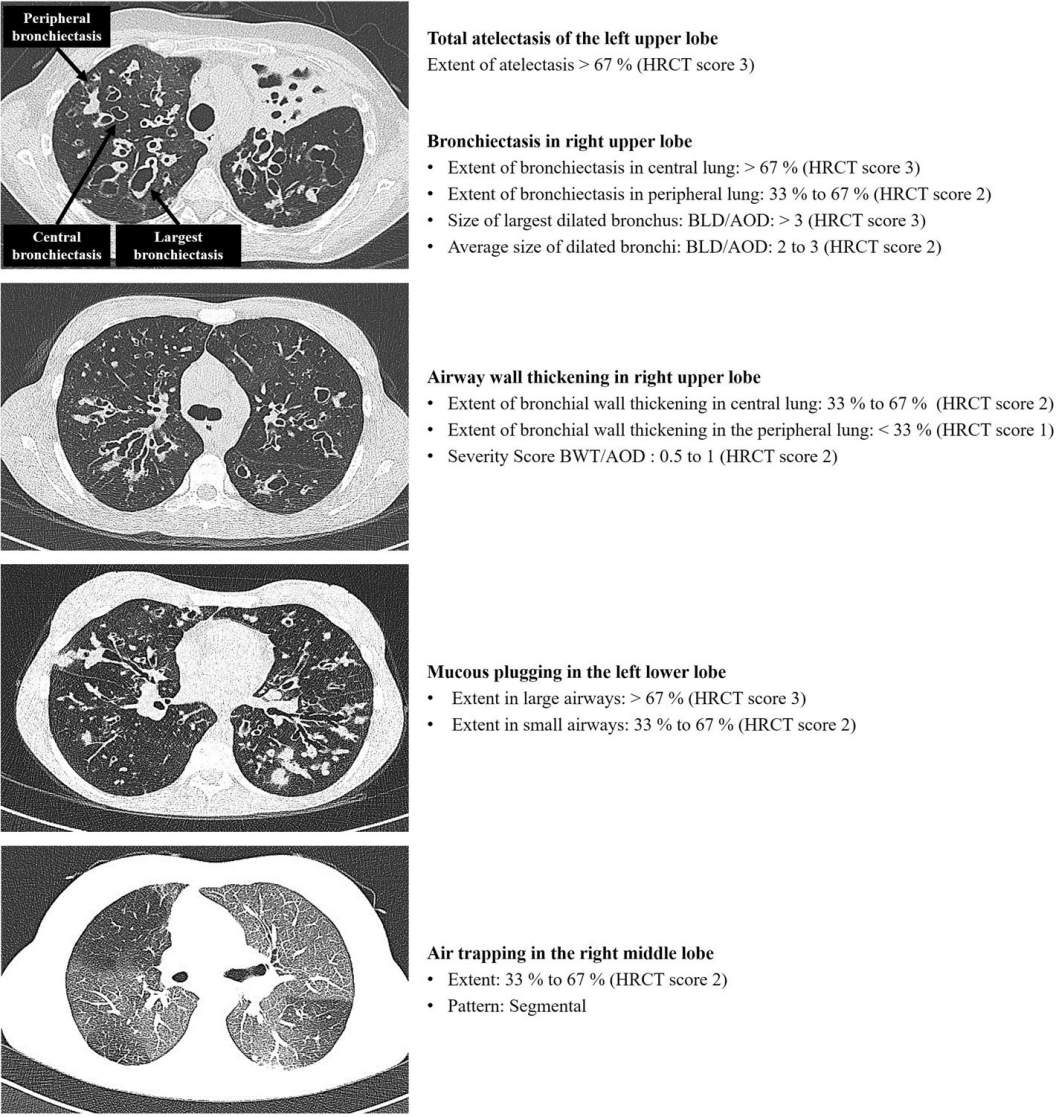
Example of HRCT metrics according to the Brody II scoring system. *Abbreviations:* BLD indicates bronchial lumen diameter; AOD, adjacent pulmonary artery outer diameter; BWT, bronchial wall thickening

**Fig. 2:**
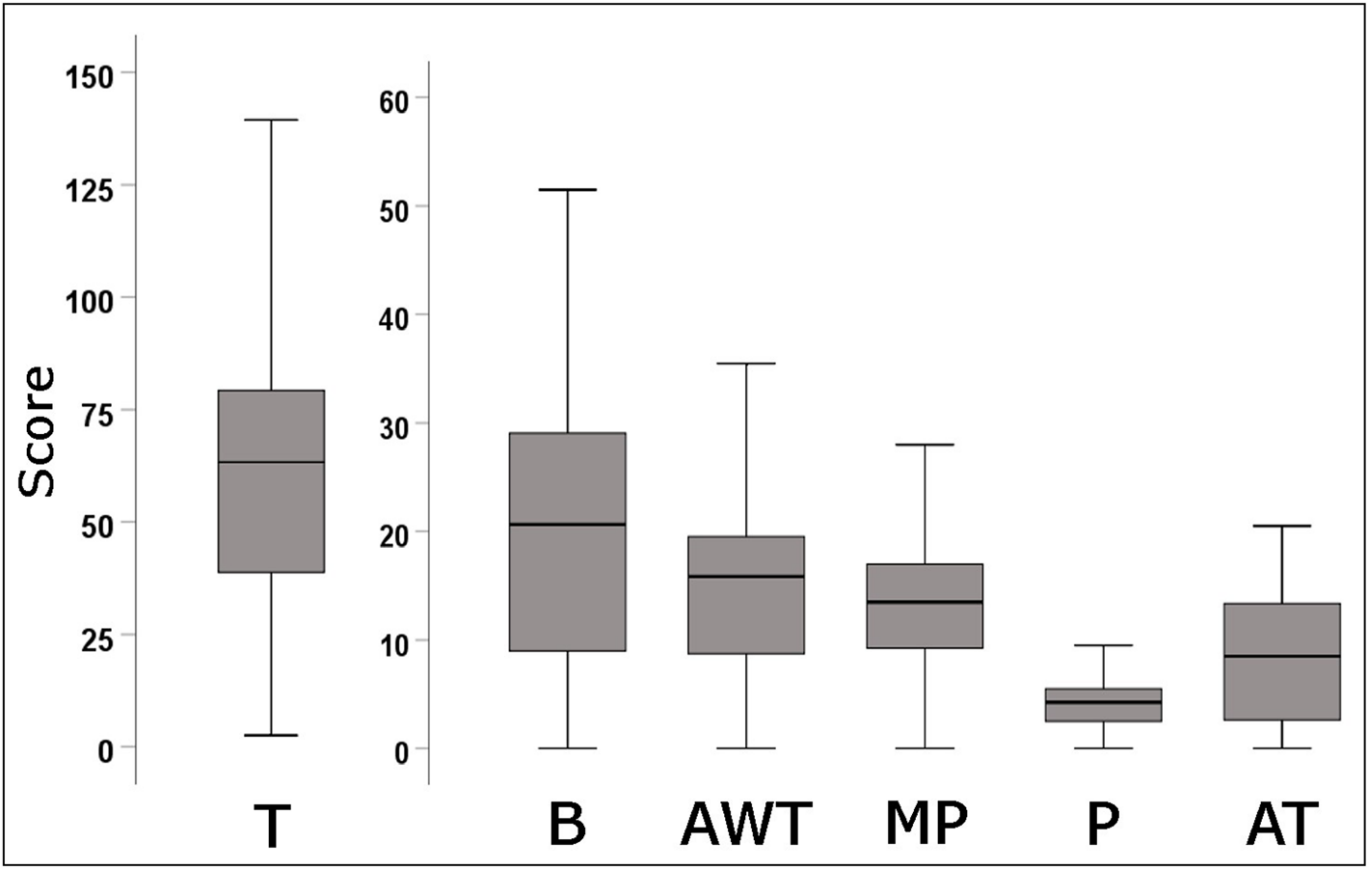
Boxplots of HRCT variables in study sample. *Abbreviations:* T indicates total score; B, bronchiectasis score; AWT, airways wall thickening score; MP, mucous plugging score; P, parenchyma score; AT, air trapping score

**Fig. 3:**
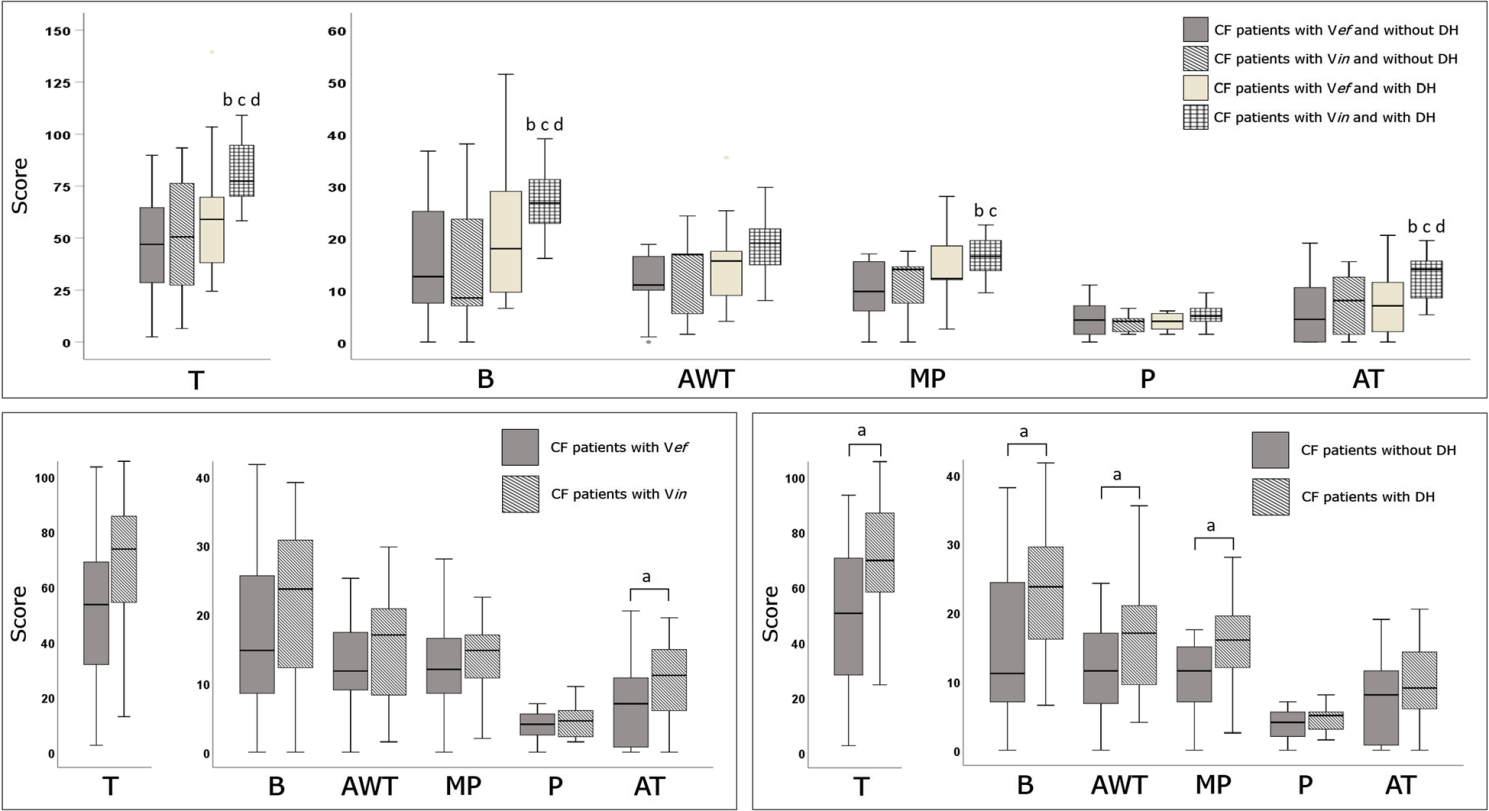
Boxplots of HRCT variables according to the presence of associated ventilatory efficiency/inefficiency and/or presence or absence of dynamic hyperinflation (*(top),* ventilatory efficiency/inefficiency only *(bottom left)*, and presence/absence of dynamic hyperinflation only *(bottom right)*. *Abbreviations:* V*ef* and V*in* indicates ventilator efficiency and inefficiency, respectively; DH, dynamic hyperinflation; T, total score; B, bronchiectasis score; AWT, airways wall thickening score; MP, mucous plugging score; P, parenchyma score; AT, air trapping score. ^a^ *p* value calculated between groups; ^b^ *p* < 0.05 versus patients with V*ef* and without DH; ^c^ *p* < 0.05 versus patients with V*in* and without DH; ^d^ *p* value < 0.05 versus patients with V*ef* and with DH

The interrater reliability between the two radiologists was “excellent” for T (ICC 0.95; 95% CI 0.91 to 0.97), B (ICC 0.93; 95% CI 0.88 to 0.96), MP (ICC 0.93; 95% CI 0.88 to 0.96), and P (ICC 0.91; 95% CI 0.84 to 0.95), while was “good” for AWT (ICC 0.86; 95% CI 0.75 to 0.92) and AT (ICC 0.81; 95% CI 0.66 to 0.88). Then, the average value of each Brody II score was considered for all analyses. Examples of HRCT metrics according to the Brody II score are provided in Fig. 1, while in Fig. 2 are illustrated for all patients the HRCT variables related to the Brody II scores. Among V*in* patients (Fig. 3, *bottom left*), only AT differed significantly compared to those with V*ef*. Patients with DH, in comparison to those without DH (Fig. 3, *bottom right*), had higher values of T, B, AWT, and MP, respectively. Patients with V*in* and with DH had higher values of T, B and AT compared to other groups (Fig. 3, *top*).

**Fig. 4:**
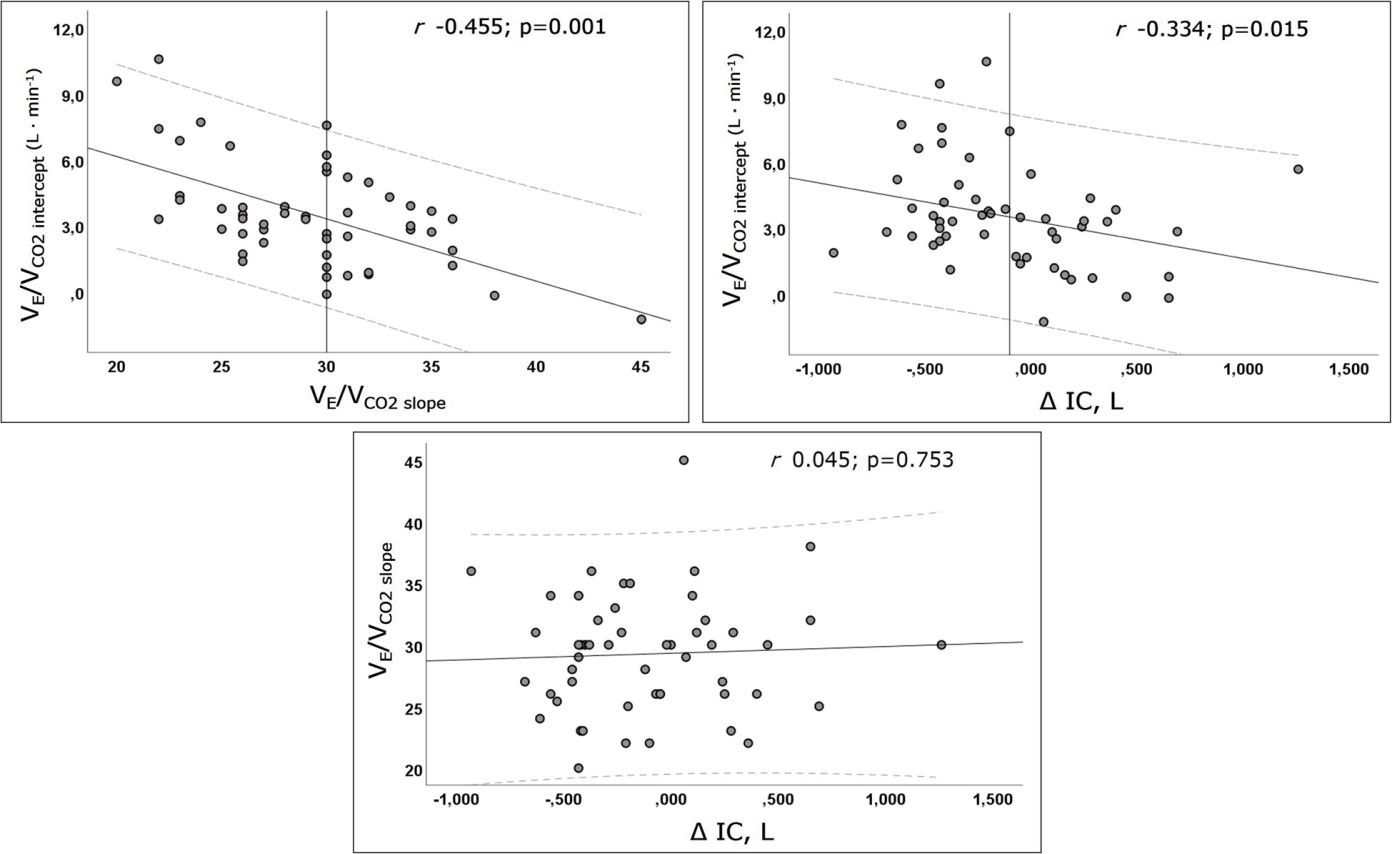
Scatterplots between V’_E_/V’_CO2 slope_, Δ IC and V’_E_/V’_CO2 intercept_. Continuous and dash lines represent fit line and 95% CI, respectively. Vertical lines in the above scatterplots represent the defined cut-off of V’_E_/V’_CO2 slope_ and Δ IC (30 and − 100 L, respectively)

**Fig. 5:**
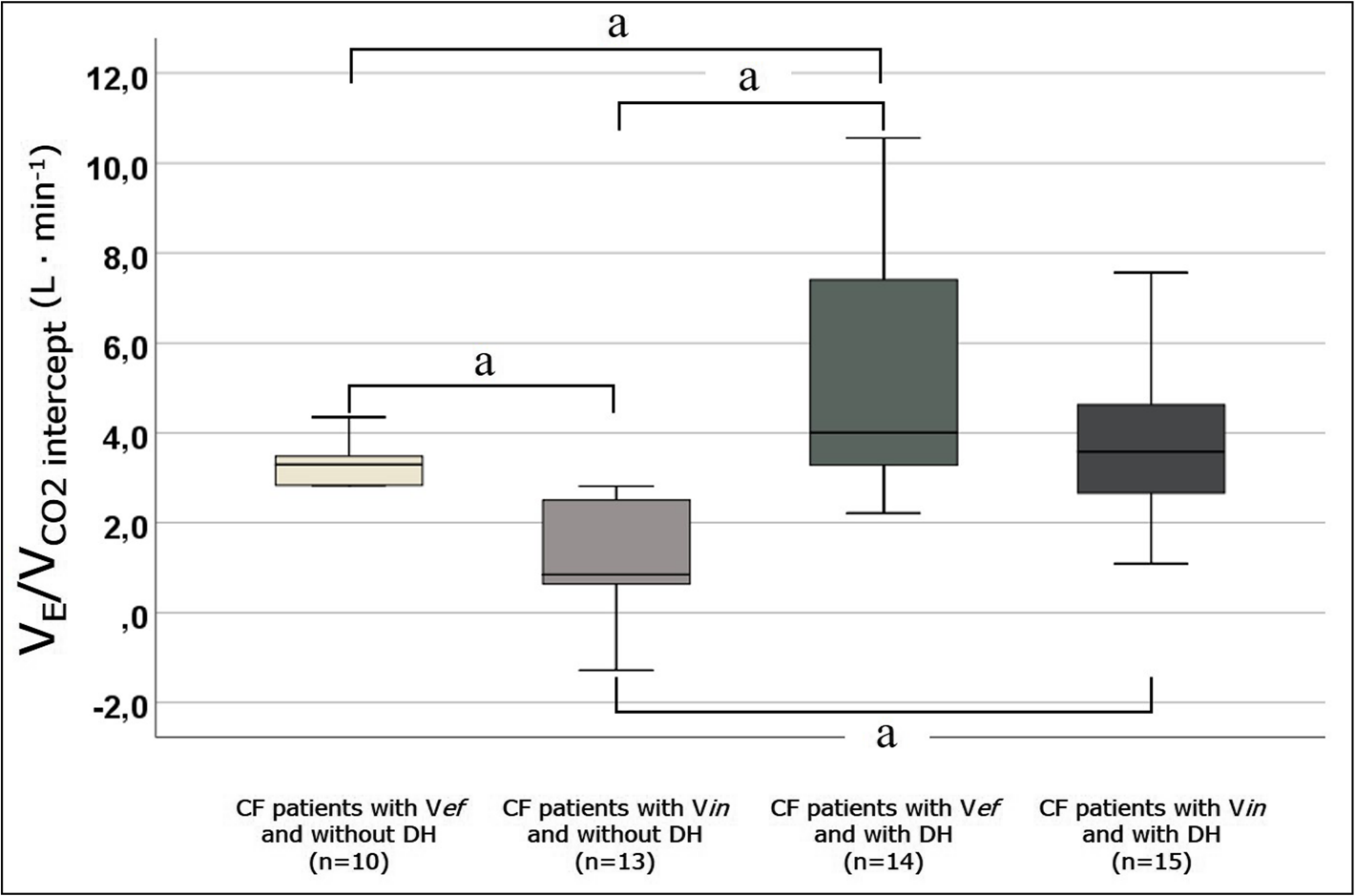
Boxplots of V’_E_/V’_CO2 intercept_ according to the presence of associated ventilatory efficiency/inefficiency and/or presence or absence of dynamic hyperinflation. *Abbreviations:* V*ef* and V*in* indicates ventilator efficiency and inefficiency, respectively; DH, dynamic hyperinflation. ^a^ *p* < 0.05

Significant correlations were found between V’_E_/V’_CO2 intercept_ and V’_E_/V’_CO2 slope_ and between V’_E_/V’_CO2 intercept_ and Δ IC. No correlation, however, was found between V’_E_/V’_CO2 slope_ and Δ IC (Fig. 4)*.* Multiple comparisons between patient subgroups (Fig. 5) showed that V’_E_/V’_CO2 intercept_ was significantly higher among patients with V*ef* and with DH compared to those with V*ef* and without DH and those with V*in* and without DH. Moreover, V’_E_/V’_CO2 intercept_ was significantly lower in patients with V*in* and without DH than those with V*ef* and without DH and those with V*in* and with DH.

Regression analysis (Table 3) showed that AT was the only independent predictor of V*in*, whilst T, B, AWT and MP were significant predictors of the development of DH.

**Table 3:** Receiver operating characteristic (ROC) variables and univariate regression predicting the probability to have exercise ventilatory inefficiency or dynamic hyperinflation

Variables	ROC variables					Univariate regression		
	Cut-off	AUC	95% CI	Sensitivity	Specificity	OR	95% CI	*p* value
Dependent variable: Ventilatory inefficiency								
AT (score)	7.9	0.682	0.535 to 0.829	0.714	0.583	3.50	1.10 to 11.09	**0.033**
Dependent variable: Dynamic hyperinflation								
T (score)	53.6	0.707	0.564 to 0.849	0.793	0.565	4.98	1.47 to 16.86	**0.010**
B (score)	16.1	0.699	0.550 to 0.848	0.759	0.608	4.88	1.48 to 16.12	**0.009**
AWT (score)	13.0	0.663	0.514 to 0.811	0.724	0.565	3.41	1.07 to 10.87	**0.038**
MP (score)	11.7	0.711	0.570 to 0.851	0.793	0.521	4.18	1.24 to 14.09	**0.021**

*Abbreviations: AT* indicates air trapping score, *T* total score, *B* bronchiectasis score, *AWT* airways wall thickening score, *MP* mucous plugging score

Boldface variables are statistically significants

## Discussion

Our study in adult patients with CF allows us to highlight two main findings: *1)* there is no correlation between V*in* and DH and variables derived from spirometry and CPET seem to be complementary and specific to one condition rather another and *2)* Brody II scores at HRCT are able to discriminate and predict V*in* (AT) and DH (T, B, AWT and MP).

### Ventilatory response to exercise in CF patients

There are very few data about the ventilatory response to exercise in adult CF patients. In a retrospective study on 102 CF patients with mild-to-moderate lung disease, the authors reported that V_E_/V_CO2 at peak_ was not related to airflow obstruction but to the V_O2 at peak_ [5]. High values of V_E_/V_CO2 slope_ identify greater dead space (V_D_) due to more extensive emphysema and/or high pulmonary vascular pressures, poorer cardiac performance, higher sympathetic drive, worse exertional hypoxemia and greater ergoreceptor stimulation [18]. Indeed, in a sample of CF children undergoing sub-maximal constant work rate exercise testing, the arterial partial pressure of carbon dioxide (PaCO_2_) levels were significantly related to alveolar ventilation rather than V_E_; this denotes the leading role of physiologic V_D_ in the quantitative determination of ventilatory response to exercise [24].

In our cohort, data about the relationship between DH and lung function impairment at rest are in line with Stevens et al. [6]. Surprisingly, we did not observe differences in term of exercise tolerance [6]; however, patients with DH and V*in* had a tendency to show a poorer exercise tolerance (Table 2). It is of note that in COPD patients, V_E_/V_CO2_ measurements may explain exercise intolerance, particularly in milder and in symptomatic patients with preserved FEV_1_ [25], since an increased V_D_ may occur [25].

### The value of HRCT metrics in V*in* and DH

HRCT scores and CPET-variables have been often used separately to predict future outcomes in CF patients, such as early structural alterations [3] or disease progression [10, 11]. However, in adult CF patients no studies looked for determinants of two exercise-induced functional scenario (presence of V*in* and DH) using HRCT scores.

Air trapping at HCRT is an important marker of distal airways disease in children with CF [26] and in COPD patients [27]. Using a modified Bhalla scoring system, a retrospective study [28] demonstrated that HRCT abnormalities were more sensible than lung function (evaluated only by FEV_1_) to detect disease decline of adult CF patients; notably, patients with air trapping and moderate-to-severely impaired lung function had more rapid rate of deterioration [28]. Moreover, in children with CF, air trapping demonstrated a significant relationship with non-invasive measures of ventilation inhomogeneity, such as the lung clearance index (LCI) [29]. These aspects are in line with our regression analysis, in which AT score independently increases the probability to have V*in* which, at least for COPD patients, may define a series of worse disease-related features [18]. Similarly, HRCT metrics on emphysema measured by low attenuation areas (LAA%) - that conceptually may be seen as a surrogate of AT - were shown to significantly explain V_E_/V_CO2 slope_ in COPD patients in a multivariate adjusted linear regression model [12].

MP, secondary to the basic ion-transport defect of the disease, represents a recognized HRCT hallmark of disease [30] and, together with perfusion abnormalities seems to play a relevant role in exacerbation [31]. Based on our results, it may also have a potentially reversible role on DH.

### The V’_E_/V’_CO2 intercept_ in CF patients

The relationship between V’_E_ and V’_CO2_ - when V’_CO2_ is equal to zero - represents the V’_E_/V’_CO2 intercept_ [18]. In normal subjects, V’_E_/V’_CO2 intercept_ is a small positive value (< 3 L∙min^− 1^) [32]. In COPD patients, V’_E_/V’_CO2 intercept_ is related to greater DH [18] and it is associated with exertional dyspnea [18].

In our CF patients, V’_E_/V’_CO2 intercept_ values lead us to some considerations. Among the whole study group, the median value was high (3.28) and, in particular, it was higher in patients with V*ef* (V’_E_/V’_CO2 slope_ < 30) and with DH. As shown for COPD patients [18], a high V’_E_/V’_CO2 intercept_ is a necessary compromise for the progressive mechanical respiratory constraints, increased V_D_ and reduced V’_E_/V’_CO2 slope_. Notably, it is proved that V’_E_/V’_CO2 intercept_ reflects some aspects about the V_D_ [33]. The increased V_D_ and ventilation inhomogeneity [29] in our CF patients may explain the increased value of V’_E_/V’_CO2 intercept_ in DH patients. Then, in this context it is plausible that V’_E_/V’_CO2 intercept_ correlates with both V’_E_/V’_CO2 slope_ and Δ IC. Because each condition (V*in* and DH) influences, in different ways, the V’_E_/V’_CO2 intercept_ (Table 2), the level of this parameter does not increase if the two conditions coexist (Fig. 5).

### Strength and limitations of the study

Although our study has some important points of strength (originality because for the first time the exercise-induced ventilatory response was considered and the wide cohort of adult CF, in which all participant performed both HRCT and CPET) we need to report some limitations. First, our study concerns data collected in a single center in Italy, in adult CF with a wide range of lung function from normal to severe airflow obstruction and a relatively well-conserved maximal exercise capacity. The considerations derived from our analyses cannot be translated into patients with different functional and physical characteristics. Secondly, data about ATs have been collected only during the inspiration phase. Although in CF a pilot study demonstrated a very good agreement between HRCT scores from end-expiratory and end-inspiratory scans [34], we cannot be sure that the execution of the end-expiratory phase scans could have changed our findings.

## Conclusion

In summary, our prospective study performed in a cohort of adult patients with CF showed the values of HRCT metrics as determinants of V*in* (AT) and DH (T, B, AWT and MP). Moreover, we highlighted in CF patients the functional and clinical potential role of V*in* and DH. We believe that in the future these aspects should be considered when exercise tolerance is assessed, such as for COPD patients. The comprehensive scenario for CF patients could be more interesting.

## Abbreviations

AOD: adjacent pulmonary artery outer diameter
AUC: area under the curve
BLD: bronchial lumen diameter
BMI: body mass index
BWT: bronchial wall thickening
CF: cystic fibrosis
CI: confidence interval
CPET: cardio-pulmonary exercise test
DH: dynamic hyperinflation
DP: double product
FEV_1_: forced expiratory volume at 1st second
FVC: forced vital capacity
HRCT: high-resolution computed tomography
IC: inspiratory capacity
ICC: intraclass correlation coefficients
O_2_ pulse: oxygen pulse
P_ETCO2_: end-tidal pressure of CO_2_
ROC: receiver operating characteristic
RR: respiratory rate
SpO_2_: oxygen saturation
TLC: total lung capacity
VAS: visual analogic scale
V_CO2_: carbon dioxide production
V_E_: minute ventilation
V*ef*: ventilatory efficiency
V*in*: ventilatory inefficiency
V_T_: tidal volume
